# Gradient Hydrogels—The State of the Art in Preparation Methods

**DOI:** 10.3390/polym12040966

**Published:** 2020-04-21

**Authors:** Natalia Zinkovska, Jiri Smilek, Miloslav Pekar

**Affiliations:** Faculty of Chemistry, Brno University of Technology, Purkynova 464/118, CZ-612 00 Brno, Czech Republic; xczinkovska@fch.vut.cz

**Keywords:** gradient hydrogels, controlled gelation, tissue engineering, supramolecular smart gels

## Abstract

Gradient hydrogels refer to hydrogel materials with a gradual or abrupt change in one or some of their properties. They represent examples of more sophisticated gel materials in comparison to simple, native gel networks. Here, we review techniques used to prepare gradient hydrogels which have been reported in literature over the last few years. A variety of simple preparation methods are available, most of which can be relatively easily utilized in standard laboratories

## 1. Introduction

Hydrogels are well-known materials formed by a hydrophilic network imbibed by water, which actually plays the role of a dispersion medium. The network is usually formed by hydrophilic polymer chains interconnected by means of either chemical or physical crosslinks. Hydrogels can contain a huge amount of water (even more than 90% by weight) while retaining their shape as a solid body. Hydrogels resemble biological environments and thus can be used in a variety of biological, biomedical, or environmental applications. For instance, as cell support or cultivation media, delivery systems, tissue substitutes, or universal sorbents. Basically, their properties are determined by the nature and structure of the parent polymeric chain, the number and character of crosslinks, and the content of water. More sophisticated hydrogel materials with tailored properties can be obtained by modifying the chain, incorporating additives, or manipulating the network structure including the formation of supramolecular assemblies. Hydrogels with gradient properties, or, more simply, gradient hydrogels, are examples of hydrogels that have been intentionally modified to achieve soft materials with specific properties.

Gradient hydrogels possess a gradual or abrupt change in one or some of their properties (physical properties mostly) when moving through a single piece of hydrogel body. These “gradient properties” may include the following: some types of mechanical properties, crosslinking density and mesh size or the porosity of the hydrogel network, chemical composition, and biochemical properties. The gradient may refer not only to a spatial characteristic but also to time or to both, giving thus a spatiotemporal property change. In contrast, conventional hydrogels can be viewed as materials with uniform properties.

The field of gradient hydrogels is progressing rapidly and admirable creativity can be seen in the variety of approaches to gradient formation. In this short review, we present an overview of the techniques used to prepare gradient hydrogels which have been reported in literature over the last few years. The techniques are divided into several groups according to the common features indicated by the group names. Some studies combined techniques from two groups but are classified under a single group selected on the basis of the main preparation principle used. The interesting question of the detection of formed gradients is out of scope of this brief review and deserves a special paper. Here we only note that the most often approaches of checking the gradient include electron and light microscopy, mechanical tests performed on several sections cut from the whole material.

## 2. State of the Art

### 2.1. Layering

Perhaps the most widely used techniques are based on the successive formation of layers of a common basic hydrogel that differ in some specific properties. The layered structure forms the space gradient in these properties usually due to their sharp change between layers.

Ko and co-workers formed three-dimensional concentrations and mechanical gradients by means of a layer-stacking technique in order to generate 3D mechanical and biomolecular gradients [1]. The gradients were created using two different types of photocrosslinkable hydrogel—poly(ethylene glycol) diacrylate (PEGDA) and gelatin methacryloyl (GelMA). The photocrosslinking process of PEGDA or GelMA prehydrogel solution containing photoinitiator was initiated by ultraviolet (UV) irradiation. First, a single layer of a prehydrogel solution whose concentrations of hydrogel precursors or soluble protein could be changed for subsequent layers was added into a mold. After UV exposure and photocrosslinking of the initial solution, the next hydrogel layer was added on top of the previous one and cross-linked analogously. Changing the concentration or volume of each discrete layer, as well as the intensity of UV exposure, resulted in different gradient profiles. The initial layers exposed to repetitive UV irradiation exhibited higher crosslinking density than the later-formed layers. The gradient here refers primarily to the concentration gradient of the bovine serum albumin used as a model biomolecule to form such a gradient. Fluorescence microscopy, mechanical testing, and scanning electron microscopy were used to confirm the gradient structure. Human fibroblasts were shown to move along the protein gradient after four days of culturing inside GelMA hydrogel. The technique is simple, does not require special instrumentation, and can be viewed as a universal procedure for photocrosslinkable hydrogels.

Mredha et al. developed a technique based on the welding of pre-stretched hydrogel thin films, during which an interfacial reconfiguration process was initiated [2]. The method was demonstrated on cellulose films. The pre-stretched films were exposed to a viscous cellulose/LiCl/Dimethylacetamide (DMAc) solution, which remained trapped on the film surface and enabled the creation of highly-ordered, oriented high-water-content hydrogel systems during the subsequent welding. The wet pre-stretched hydrogel films were placed in co-facial contact under slight pressure. The thin layer of cellulose/LiCl/DMAc solution located on the interface of the two thin films induced interfacial reconfiguration of the cellulose chains. With time, the interfacial LiCl steadily diffused away creating a concentration gradient from the interface to the bulk, while the cellulose macromolecular chains reassembled due to the exchange of intermolecular H-bonds, resulting in the welding of the thin films. Four types of anisotropic multilayer hydrogels with variously oriented architectures could be prepared: parallel lamination, orthogonal lamination, axial rolling, and concentric rolling. In addition to good mechanical properties, these hydrogels exhibited asymmetric shape deformation and anisotropy in electrical conductance. The authors suggest that their welding technique should be applicable for the fabrication of hierarchical hydrogel structures using not only cellulose, but also other different kinds of supramolecular polymers. The technique is relatively simple, requiring some pre-stretching pressurizing (welding) facility, which could be very simple and realizable in a standard laboratory. The crucial point is the appropriate selection of the interface-wetting solution, which forms the concentration gradient and induces reorientation and interaction changes in the polymeric chains forming the hydrogel.

A freeze-drying method called “iterative layering” was described by Shi et al. [3]. The technique was developed primarily to fabricate gradient hydrogels that adequately mimicked the gradient structure of natural bone tissues. Their hydrogels were based on dopamine-modified alginate and quaternized chitosan-templated hydroxyapatite (QCHA). Solutions of these two precursors with appropriate concentrations were mixed and the subsequent freezing of this mixture created the first layer of a multi-layered system. The next two layers, with decreasing QCHA concentrations, were added onto the surface of the previous layer using a similar protocol as for the first layer. Then, a freeze-drying procedure followed. The final material was then immersed in a calcium ion solution for crosslinking. The gradient structure was achieved by the freeze-drying process and by changing the QCHA amount in each layer. All prepared samples showed a high degree of pore interconnectivity throughout the structure, as well as a gradient change in pore size formed especially during the freeze-drying step. The prepared hydrogel, when used as scaffolds, offered good biocompatibility, good adsorption properties, and supported cell growth and proliferation. The technique is similar to that used by Ko and co-workers [1] but requires a freeze-dryer and thus can also be more time-consuming. Otherwise, the technique is quite general and not limited only to the studied system and application.

A specific layer of nanohydroxyapatite and glycosaminoglycan was formed by Radhakrishnan et al. on alginate/poly(vinyl alcohol) (PVA) semi-interpenetrating network hydrogel [4]. The resulting gradient material could mimic the native interfacial architecture of osteochondral tissue in subchondral and cartilage layers. Therefore, this type of scaffold was fabricated with a smooth transitional gradient interface and with spatial diversity in its microstructure, composition, and mechanical and biological properties. The subchondral layer was prepared by first suspending nanohydroxyapatite in an alginate-PVA blend solution, whereas for the chondral layer chondroitin sulfate nanoparticles were used. These two pregel solutions were crosslinked with calcium sulfate. The different hydrogel layers were placed one on top of the other, crosslinking with each other to form hydrogel with a gradient interface. Subsequently, the hydrogel was lyophilized. The gradient was thus formed across the interface during the final crosslinking. The resulting multilayered gradient hydrogel scaffold exhibited an interconnected porous structure, while the co-culture of osteoblasts and chondrocytes in gradient hydrogels showed the layer-specific retention of cells and cell-cell interactions at the interface. The method is simple and applicable for the creation of interfacial gradients—the crosslinking interface must allow sufficiently slow crosslinking in order to enable gradient formation by diffusion and the interpenetration of components of the gelling matrix.

**Figure 1:**
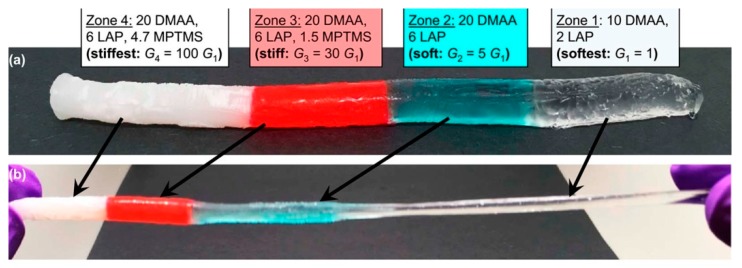
Variations of the soft−stiff hybrids: (**a**,**b**) multizone cylinders. (**a**) The gel has four zones, with zone 1 on the far right being the softest, with a shear modulus G1 ≈ 1 kPa and zone 4 on the far left having a modulus ~100 G_1_. Between these, there are two more zones, each colored with a different dye and having intermediate moduli, as indicated. Zones 1 and 2 are prepared without methacrylated silane (MPTMS) and are transparent, whereas zones 3 and 4 have MPTMS and are opaque. (**b**) The cylinder is stretched to show that zones 3 and 4 are negligibly extended, zone 2 is extended as it, and zone 1 is extended a lot. Reprinted from [5] with permission.

Multi-layered hydrogels with a gradual change in stiffness were described by Gharazi et al. [5]. The key feature of their preparation process was to use different types of nanoparticles as additional cross-linking agents in an acrylic-based hydrogel. The authors fabricated hydrogels with a smooth mechanical gradient from soft to stiff zones by varying the cross-link density between both polymeric components and nanoscale fillers contained in the hydrogel matrix. For the soft zone laponite was used, while laponite and in situ-generated silica nanoparticles were used for the stiff zone. Another important aspect was to bring the pregel solutions into contact while their viscosities were significantly high in order to avoid the undesirable mixing of zones. Thus, the first pregel solution was placed into a tubular mold and polymerized until it became sufficiently viscous, and then the second pregel solution was over-layered. This procedure was able to be repeated and the authors demonstrated gels with up to four well-defined distinct zones with clearly defined interfaces between them. Variations of the soft-stiff hybrid hydrogels is illustrated in Figure 1.

Covalent linkages of polymer chains at each interface ensured the robustness and strength of the zone junction. The fabricated multilayer systems were flexible and stretchable and offered a significant contrast in mechanical properties between the soft and stiff zones. The proposed gels could be used in tissue engineering for connecting implants with underlying tissue. This method is similar to that used by Ko and co-workers [1] and is based on changing the composition of an identical gel basis, thereby giving different crosslinking densities and allowing controlled over-laying, which should prevent the mixing of different compositions but still enable the creation of chemical junctions between them.

The aim of the work by Scaffaro et al. was to prepare a gradient pore structure in the basic polylactide (PLA) matrix [6]. An eco-friendly porogen technique was employed which did not need the use of organic solvent. Water-soluble NaCl and polyethylene glycol (PEG) were used as water-leachable porogens. Initially, PLA/PEG/NaCl blends with different NaCl crystal sizes were prepared by melt mixing using a laboratory blend mixer. The three blends were subsequently compression molded in appropriate molds in three layers using a laboratory press at an elevated temperature (210 °C). Finally, the porogens were cleared away by leaching in boiling demineralized water. A pore size gradient was achieved due to the NaCl incorporation of different grain sizes, while PEG solvation provided micropores in hydrogel structures. The obtained hydrogels were proposed for use as scaffolds in the field of bone regeneration. The technique is an example of combining traditional methods with the instruments of plastics processing to create a (pore-size) gradient structure.

### 2.2. Electro-Based

An electrical field can be used to move charged entities in the originally homogeneous system during crosslinking. The movement creates a spatial gradient along the field axis, which is fixed by the crosslinking process. These techniques are simple and except for a source voltage and electrodes no special equipment is needed in comparison to the conventional preparation of hydrogels. Of course, an electro-based approach can be applied only when electrically movable components are present.

**Figure 2:**
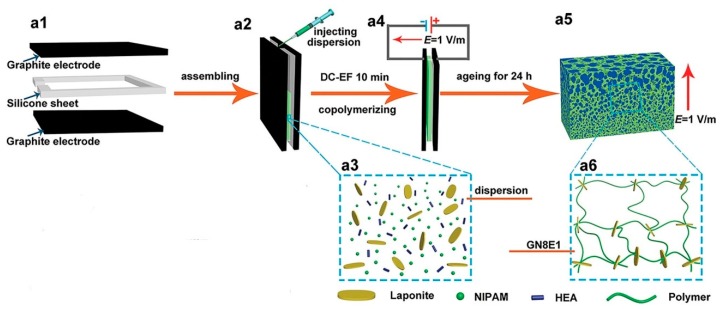
Scheme of synthesis of the bionic gradient hydrogel (**a1**–**a4**), polymerization dispersion (**a5**), and gradient network structure (**a6**). Reprinted from [7] with permission.

Tan et al. used an electrophoresis technique to move charged components, thus creating a gradient structure [7]. Their hydrogels were prepared by copolymerizing hydroxyethyl acrylate and N-isopropylacrylamide monomers. Negatively charged laponite particles were dispersed in the monomer solution and used as electrically active components. Gelation was realized between two graphite electrodes separated by a silicone frame spacer. During the copolymerization of the monomers, a direct current electric field was applied to the electrodes, which caused the charged laponite particles to move towards the anode, resulting in a gradient gel structure. Scheme of synthesis of the bionic gradient hydrogel is shown in Figure 2.

The resulting hydrogels were intended as actuators in air in fields such as soft robotics and the use of artificial muscles in various environments. The scanning electron microscopy of freeze-dried samples demonstrated the long-range gradient network structure along the direction of the electric field, which was formed by the crosslinking effect of laponite. The average network dimension was found to increase from 0.35 μm on the anode side to 4.83 μm on the cathode side.

The same technique was successfully used by the same group also for the creation of laponite-containing poly(N-isopropylacrylamide)-based [8] or laponite-containing hydrogel formed by copolymers of the thermo-responsive monomers N,N-diethylacrylamide and (2-dimethylamino) ethyl methacrylate [9].

Another version of electrophoretically induced gradients was presented by Yang et al. [10]. In their work, the electrophoresis was combined with microphase separation to obtain a color-changing hydrogel actuator. A gradient was created to ensure a fast, temperature-triggered complex deformation function for the actuator. Hydrogel was prepared by the polymerization of N-isopropylacrylamide and [2-(methacryloyloxy)ethyl]trimethylammonium chloride (DMC). As a cationic comonomer, DMC can migrate toward the cathode under an electric field and form a gradient distribution after copolymerization in the final hydrogel. A gradient distribution of DMC was thus formed during polymerization due to the migration of DMC under the electric field. Microphase separation which occurs between homopolymers of N-isopropylacrylamide and its copolymers with DMC during the process of polymerization was employed to form a hierarchical hydrogel structure.

**Figure 3:**
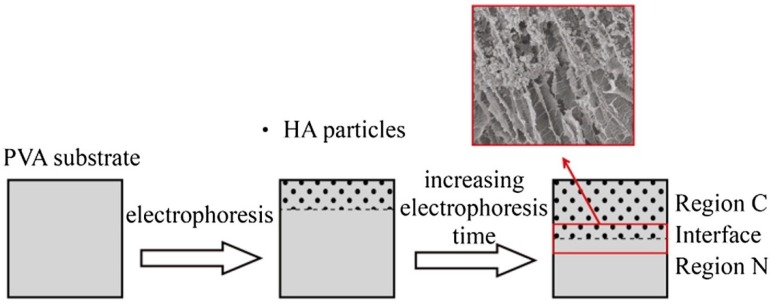
Illustration of the electrophoresis process. Insertion is the scanning electron microscope (SEM) image of the Interface. Reprinted from [11] with permission.

The electrophoresis method was combined by Su et al. with directional freezing-thawing to synthesize bilayered poly(vinyl alcohol) (PVA)/hydroxyapatite composite hydrogels with anisotropic and gradient mechanical properties [11]. Hydroxyapatite nanoparticles were used to overcome the very low cell adhesion on PVA hydrogels, which otherwise can be widely used for biomedical application, especially in the field of tissue engineering. Further, the nanoparticles enhance the mechanical strength of the hydrogel scaffold. Initially, PVA hydrogels were prepared by the directional freezing-thawing, which created an aligned channel structure. This was achieved using liquid nitrogen, which formed a temperature gradient inside the hydrogel, thus inducing the growth of ice crystals along the temperature gradient and pushing the PVA molecules aside. During the following thawing process, PVA chains formed crystallites, which provide the structural stability, as well as the elasticity of the hydrogel. Additional directional freezing–thawing cycles induced a further increase in channel size. Subsequently, during electrophoresis in calcium chloride and disodium hydrogen phosphate solution, hydroxyapatite particles were formed within the channels and distributed uniformly. By controlling the duration of the electrophoresis procedure, a bilayered hydrogel with half-composite and half-non-composite regions could be synthetized. Illustration of the electrophoresis process published by Su et al. is illustrated in Figure 3.

The pore structure of the formed bilayered hydrogels resembled the anisotropic construction of natural articular cartilage. The technique is rather elaborate but still does not require special equipment—the freezing was realized by the simple immersion into liquid nitrogen of the PVA precursor in a mold. A pore-mechanical gradient was created by this simple freezing process; thus, it is probably difficult to exercise precise control over the resulting gradient.

**Figure 4:**
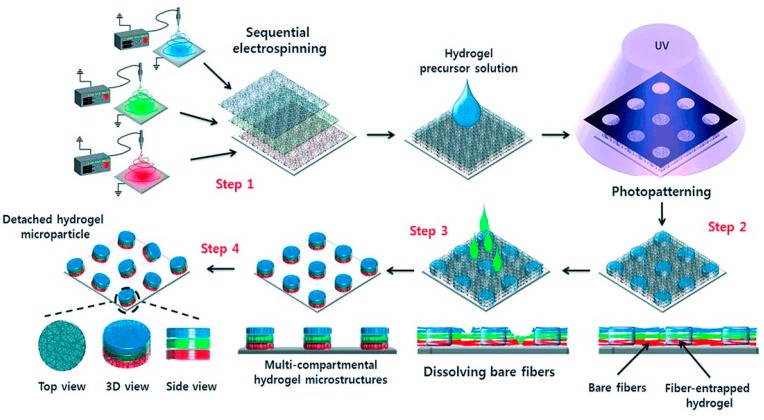
Schematic illustration of preparing multi-compartmental hydrogel microparticles by using sequential electrospinning and photopatterning. Reprinted from [12] with permission.

Cho et al. described a combination of electrospinning and photopatterning as a technique for fabricating multi-compartmental non-spherical hydrogel microparticles [12]. The electrospinning was realized as a sequential process which allows the production of multi-layered fiber hydrogel scaffolds with distinct compositions in each layer, which is basically a compartment of the microparticle. The whole procedure is illustrated in Figure 4.

In the first step, a fiber matrix containing a specific component was electrospun until a predefined thickness was obtained. In the second step, another fiber matrix containing a different component was placed on top of the first matrix. This process may be repeated until the desired number of fiber matrices are obtained. Then, the photo-active fiber components in the multi-layered fiber matrix were photopolymerized under exposure to UV light through a photomask creating two different microdomains. Each microdomain consisted of a bare fiber region and a fiber-entrapped hydrogel region. The bare fibers were selectively removed with hydrophobic chloroform to produce an array of hydrogel microstructures entrapping the multi-layered fibers. Finally, by scraping the hydrogel microstructures from the substrate, the resultant multicompartmental microstructures were obtained, while each fiber matrix became a compartment of hydrogel microparticles with different vertical functional properties. Each compartment of a microparticle is composed of a fiber matrix with a different composition. The technique is rather elaborate and, in addition to a UV source and photomask, requires an electrospinning facility.

### 2.3. Controlled Gelation

This represents a large variety of techniques in which the gelation process is controlled in space. The control is achieved by controlling the concentration of hydrogel precursor(s) or the gelling environment like pH, temperature, or intensity of irradiation in the case of light-cured materials.

Fan et al. suggested the preparation of dual-gradient hydrogel materials, i.e., hydrogels with chain and cross-linking density gradients [13]. The dual-gradient should mimic fast actuation phenomena in nature such as the snapping leaves of a flytrap. The gradient should deliver the capacity to accumulate elastic energy and rapidly release the energy through ultrafast (snapping) deformation. The authors used UV-induced free radical generation on graphene oxide to control the polymerization of hydrogel precursors (a mixture of N,N-dimethylaminoethyl methacrylate and N,N′-methylene-bis-acrylamide). UV irradiation is strongly absorbed by graphene oxide and leads to the formation of a light intensity gradient along the irradiation axis. A higher UV intensity causes the generation of a higher concentration of free radicals on the surface of graphene oxide and thus accelerates the polymerization of the monomers in comparison to regions subject to lower intensity. Thus, in the abovementioned study, the UV-exposed side had both higher chain and cross-linking densities than the other side. This is a simple and elegant method applicable for hydrogels formed by radical polymerizations; the precise control of gradient formation might be a challenge because such formation is based only on the penetration depth of UV irradiation. For the same reason, this technique is probably applicable only to the production of thin hydrogel sheets (the mold used in the original source was 0.5 mm thick).

Another procedure for the control of UV-initiated gelation was published by Kim et al. [14]. A linear stiffness gradient in hydrogels based on gelatin methacryloyl was formed with the aid of photomasks that limited the penetration of UV light; thereby, different degrees of polymerization were initiated in the gelling material. Gelatin methacryloyl prepolymer was placed between a coverslip and a microscope slide, forming a sandwich structure, and UV irradiated through a photomask of 20%–100% transparency. The mask was printed on a transparent film and enabled different light penetration across 15 mm of length. The stiffness gradient across the 15mm hydrogel ranged from about 4 to 13 kPa as determined by atomic force microscopy (AFM). The method is applicable perhaps for any light-cured hydrogels, the mask enabling rather precise control of the curing process and, consequently, of the formed gradient.

**Figure 5:**
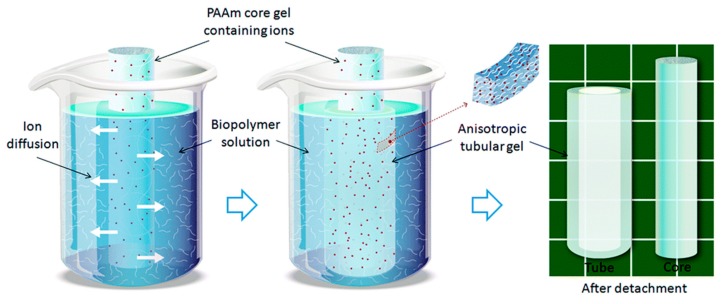
Design strategy. A cylindrical polyacrylamide (PAAm) core hydrogel loaded with small diffusible ions/molecules was inserted into a biopolymer solution. If the polymers self-bind in the presence of ions/molecules, a self-grown anisotropic hydrogel tube will form on the core hydrogel surface by radial diffusion. The resulting tube can easily be detached from the core due to the non-adhesive behavior of both hydrogels. Reprinted from [15] with permission.

Mredha et al. suggested the fabrication of anisotropic tubular hydrogels by the controlled diffusion of small ions as gelators [15]. The method is shown in Figure 5.

First, the core hydrogel containing ions for the subsequent gelation is prepared in a cylindrical shape by a standard procedure (polyacrylamide was used by the authors). The core hydrogel is immersed into a biopolymer (alginate) reservoir solution capable of inducing gelation by means of the presence of small ions (Ca^2+^, Cu^2+^, or Zn^2+^ in the case of alginate). The small ions diffuse from the core hydrogel through the alginate solution and cause gelation of alginate. At the same time, the concentration gradient of the diffusing ions results in a “crosslinking gradient” of alginate. After a specified diffusion time, the whole sample is removed from the alginate solution and immersed in water to remove non-gelled biopolymer. After washing, the core hydrogel is pulled from the surrounding hydrogel tube. This hydrogel tube containing gelled alginate is stabilized by immersing it overnight in an ion solution of the same initial concentration. The final result is a tubular hydrogel with a gradient wall thickness. Several process parameters enable control not only of the gelation process but also of the shape of resulting gradient hydrogel. These include the concentration of small ions or biopolymers, the diffusion time, the flow direction of the ions, and the size and shape of the core hydrogel. In this way, the authors prepared various types of complex tubular-shaped hydrogels with well-modulated morphological properties. The viscoelastic properties of the prepared hydrogels were highly tunable and relatively similar to those of native blood vessels. The method is simple and applicable to systems in which no extreme differences in the rates of diffusion and crosslinking exist.

Liu et al. prepared hydrogels with gradient chemical structures using modified microcrystalline cellulose as a precursor [16]. Cellulose was oxidized by sodium periodate to prepare its dialdehyde derivative. Gelation was achieved by the introduction of ammonia gas into an aqueous solution of dialdehyde cellulose and various diamines. The gas was aerated into a solution containing 1,6-hexanediamine dihydrochloride for a certain time until a certain volume of the original solution became hydrogel. At this moment, the gas flow was immediately stopped and an aqueous cyanamide solution was added to the surface of the hydrogel. Then, ammonia gas was aerated into the solution once more until the remaining solution turned into the hydrogel. The addition of the second diamine ensured the creation of a gradually changing hydrogel structure, which was thus fabricated by controlling the chemistry of the gelling components. The method as described is directly applicable for hydrogels made by reactions between aldehyde and amino functionalities and is simple to perform, though the use of ammonia gas may be unpleasant.

The fabrication of chitosan-based hydrogels with gradient structures via a step-by-step controlled crosslinking process was reported by Xu et al. [17]. Sodium tripolyphosphate and sodium hydroxide were used sequentially as cross-linkers of the positively-charged biopolymer chitosan. The gelation process as well as the morphological properties of the final product were modulated by varying the gelling conditions such as gelling time, pH, and composition of the reaction media. First, chitosan solution was frozen to a desired shape. The frozen chitosan was immersed consecutively in two gelling solutions, first in a tripolyphosphate and sodium chloride solution at 4 °C for one hour, during which time complete gelation was observed. The formed hydrogel was then further treated by a second gelling solution—sodium hydroxide—for 10 min at most. The final product was washed in de-ionized water or PBS (pH = 7.4). The gelation process in the first step occurred gradually from the surface; after 1 h, the whole sample had transformed into gel but its interior gel-like character resulted from the ionic strength-dependent solubility of chitosan. The gelation process was completed in the second gelling solution. The resulting hydrogel exhibited a three-layer microstructure. This technique employed chitosan’s specific pH-dependent solubility and dissociation together with its ionic crosslinking. However, diffusion-controlled crosslinking using one frozen (i.e., solid) gelling component immersed into a second liquid gelling component at lowered temperature could be applicable more generally.

A hydrothermal procedure was designed by Luo et al. for the synthesis of gradient porous elastic hydrogels with shape-memory properties [18]. A heterobifunctional crosslinker was employed, which enabled the use of a hydrothermal process in the synthesis. N-isopropylacrylamide, a thermo-responsive material with two highly reactive double bonds, was used as a hydrogel network forming monomer. 4-hydroxybutyl acrylate was used as heterobifunctional crosslinker, which possesses reactive double bonds and less reactive hydroxyl groups. N-isopropylacrylamide was firstly polymerized by 4-hydroxybutyl acrylate via hydrothermally induced free radical vinyl polymerization, during which polymer chains with pendant hydroxyl groups (PNIPAM-OH) were formed. This product precipitated while creating a diminishing concentration gradient of PNIPAM-OH from the top to the bottom of the hydrothermal reactor. The precipitation process was accompanied by intermolecular dihydroxylation, occurring via hydrothermally induced dehydration polymerization, resulting in gradient porous hydrogel. This technique is based on specific reactions under the conditions of a hydrothermal process at elevated temperature, such as vinyl polymerization and intermolecular dehydration, and is thus amenable to hydrogel precursors of corresponding chemical composition. The mechanism of the gradient formation during precipitation is not fully clear.

Gorgieva and Kokol combined spatiotemporal and temperature-controlled gelation with a freezing-lyophilization process to fabricate hydrogels with pore size gradients and improved thermomechanical resistance [19]. Cryogelation was the principle technique used to form the porous structure. During cryogelation, a polymeric precursor is displaced into a non-frozen microphase and polymeric gel is concentrated within the pore walls. The procedure was realized in a simple custom-made arrangement including a plastic Petri dish with polymeric solution placed on a Cu-plate temperature-controlled by a Peltier element (at –12 or –16 °C). Hydrogels were based on gelatine crosslinked by 1-ethyl-3(3-dimethylaminopropyl)-1-carbodiimide hydrochloride (EDC) and N-hydroxysuccinimide (NHS). First, cryogelation of the gelatine solution was performed and the resulting material was post-crosslinked after its lyophilization by means of incubation in EDC/NHS solution. By modulating the reaction media (PBS buffer solution and a 1:4 PBS/ethanol mixture) and employing different molarities of reagents and variable time extensions (1 h till 1 day), the authors were able to prepare tunable cryogels with gradient microporosity. The addition of ethanol in the reaction media had an enormous influence on the morphological properties of the gels (round particles in PBS to ellipsoid particles in the PBS/EtOH mixture), which consequently reduced the swelling properties to some 50% of the values obtained with 100% PBS. The porosity gradient profile arises from the cryogelation parameters. Changing these parameters resulted in different degrees of crosslinking (e.g., the presence of 100% PBS and a higher EDC concentration resulted in an approximately 40% degree of crosslinking). The crosslinking degree is directly proportional to the porosity of the cryogels. Cryogelation can be viewed as a general method to create porosity gradients, providing the rates of phase separation and network formation are well balanced. As shown in the abovementioned paper, the required cryogelation equipment can be assembled relatively easily in a standard laboratory.

**Figure 6:**
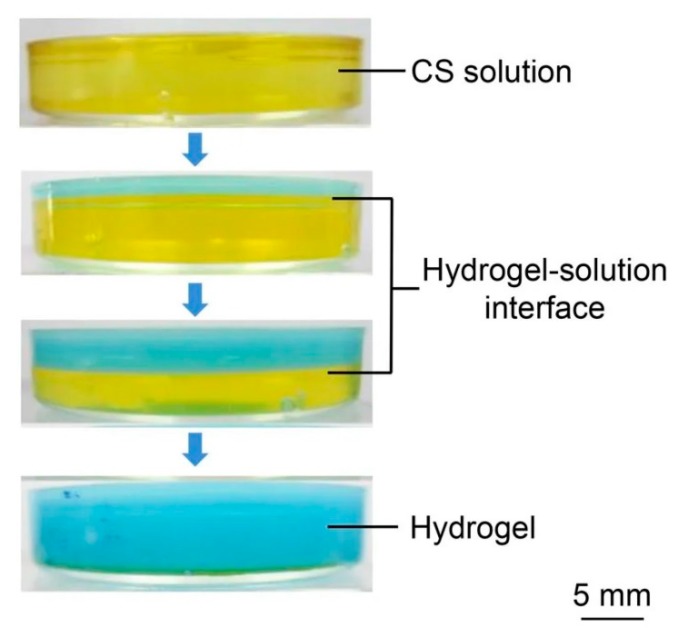
Preparation of chitosan hydrogel by a single opening mold. Digital photographs of gelation process at different reaction time. Solution and hydrogel were colored by bromothmol blue for visibility. In view of pH range, the blue part corresponded to hydrogel already formed while the bellow part corresponded to unreacted solution and the boundary indicated the hydrogel-solution interface. Reprinted from [20] with permission.

Multi-layered chitosan hydrogel with an oriented structure was prepared by controlling the gelation process for chitosan (Figure 6) [20]. No additional crosslinking agent was used and the control of the process was based on the specific pH-dependent solubility and gelation of chitosan. The technique is based on the gelation process for chitosan dissolved in an acidic aqueous solution which comes into contact with alkali. Instead of forming cross-links in the whole solution in a single-opening mold, hydroxyl ions diffuse into the system through the contact interface and generate an oriented gradient structure along the path of diffusing ions.

In Nie et al.’s study, Hydrogels were prepared with different concentrations of chitosan (0.1–4.0 wt.%) and gradient structures appeared only in materials with relatively high chitosan concentrations. The technique can be used for any system in which the diffusion of the crosslinking agent is not silenced by a too-fast gelling reaction. Precise gradient control is not easy as the formation of the gradient is a natural result of diffusion combined with the gelling reaction.

### 2.4. 3D Print

Modern and rapidly developing 3D printing technologies have also entered the hydrogel arena. Liquid hydrogel precursor or precursors are delivered from a controlled-moving printing head and should solidify sufficiently quickly to provide and maintain the desired shape. Control of precursor composition or of precursor combinations during 3D printing can be used to create gradients. The fabrication of a step-gradient nanocomposite hydrogel was reported by Motealleh et al. [21]. Hydrogels were generated by the spatial connection of different nanocomposite hydrogel pastes, in which the concentration of nanomaterials was modulated by means of the 3D printing technique. The authors claim that biomaterials with both mechanical and chemical gradients have been developed with the aim of influencing cell migration, proliferation, and differentiation to regenerate tissues and tissue interfaces. Specifically, periodic mesoporous organosilica nanoparticles were coated by poly-D-lysine to obtain biopolymer-coated silica-based nanomaterials. These nanoparticles were inserted in different concentrations into alginate/laponite pre-hydrogel pastes. Laponite improved the mechanical properties of alginate dispersion and generated injectable pastes. The resulting pastes were subsequently processed by a standard 3D printing technique. Printing was controlled to create a side-by-side orientation in the form of three connected hexagonal prisms in the horizontal plane. The final construct was crosslinked with a solution of calcium ions and freeze-dried. The step-gradient was achieved by using pastes with two different concentrations of the mesoporous organosilica nanoparticles during printing. 3D printing appears to be a promising technique particularly for the creation of gradients in printed hydrogel materials when a 3D printer is available. Of course, the technique depends on the availability of printable, liquid-like hydrogel precursors which exhibit sufficient crosslinking rates once printed structures are formed. 3D printers combining several streams upon printing, either sequential of mixed, should be advantageous for the fabrication of gradient hydrogels.

### 2.5. Microfluidics

Microfluidics is another contemporary and advancing technology intended for a variety of applications including the processing of materials. A fluid hydrogel precursor moves in narrow (micrometer-sized) channels and can be mixed with a high control with another precursor. Precise control of mixing enables preparation of and manipulation with gradients in a broad range. Due to its micro-dimensions, microfluidics is employed only in laboratory studies and its expansion into large-scale production is uncertain. It will probably be restricted to the production of special products in smaller batches.

Cross et al. presented a relatively simple microfluidics preparation of gradient hydrogels which could be used as scaffolds [22]. Their hydrogels were based on two natural polymers which had been chemically modified to confer photo-crosslinking properties: gelatin methacryloyl and methacrylated kappa carrageenan. These precursors were reinforced with two dimensional nanosilicates to mimic the native tissue interface. Microfluidics was realized in a Teflon mold containing three rectangular wells. Both prepolymer solutions of equal volume were pipetted into either side of the well simultaneously. The gradient was created by counter-flow mixing in the mold due to capillary action. Upon exposure to UV-irradiation, the prepolymer mixture was covalently crosslinked and the gradient was fixed. The crosslinking of the polymers was achieved by UV exposure in order to obtain a covalently crosslinked network. The final properties of the gradient hydrogel could be tuned by optimizing the processing parameters and conditions such as: prepolymer volumes or the optimal time prior to crosslinking to allow diffusion across the prepolymer interface. The main advantage of microfluidics can be seen in the possibility of precisely manipulating the flow of liquid hydrogel precursor and thus controlling the final gradient. This was demonstrated by Xin et al. [23], who created physicochemical gradients in microporous particle hydrogels. Two different gel precursor solutions were mixed in a microfluidic device and droplet generator module. The two solutions were pumped into two different inlets of a Y-shaped microfluidic mixing module. Their flow rates could be independently adjusted by controllable syringe pumps. By changing the flow rate ratios while keeping the total flow rate constant, a stream of mixed solutions having different properties could be generated. These mixed solutions entered a T-junction droplet generator module, where the solution was pinched off by the continuous oil phase to generate discrete water-in-oil emulsion droplets. The mixing of gel solutions within the droplets was accelerated by chaotic advection within the droplets created in a winding channel through which the fluid flowed after the T-junction. The droplets then flowed into tubing and were photopolymerized downstream before the polymerized microgels were collected layer-by-layer in a syringe.

### 2.6. Controlled Fluid Mixing

**Figure 7:**
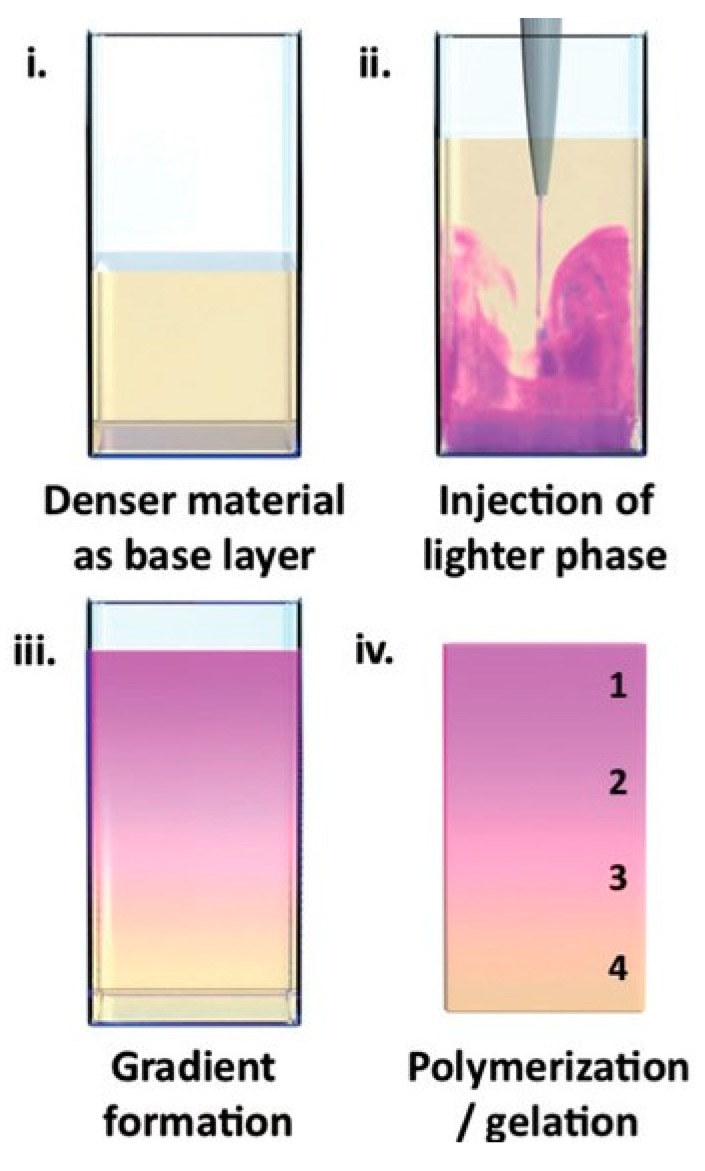
Buoyancy-driven gradient casting. Schematic showing the steps of gradient formation: (**i**) an ase layer (yellow) is added to a mold, (**ii**) the injection phase (purple) is introduced during a single injection at a controlled rate, (**iii**) the system is allowed to equilibrate and form a material gradient, and (**iv**) the gradient is preserved by gelling or polymerizing the material. Reprinted from [24] with permission.

**Figure 8:**
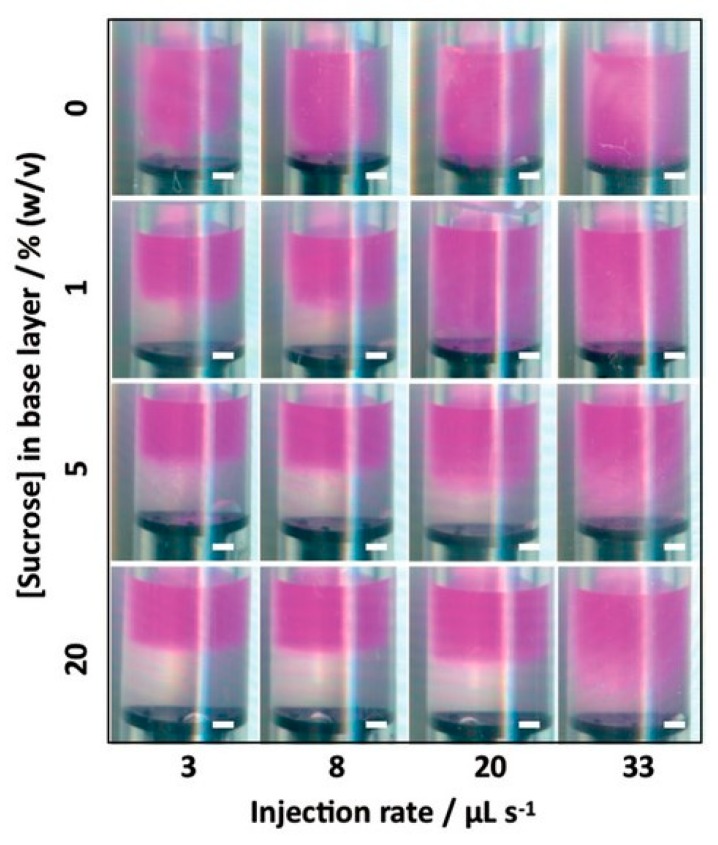
Buoyancy-driven gradient casting. The gradient pattern could be tuned by varying the injection rate and concentration of sucrose in the base layer. Reprinted from [24] with permission.

Controlled fluid mixing is, in principle, similar to microfluidics but realized on a macroscopic scale with conventional equipment and procedures. Again, liquid hydrogel precursors are mixed in a controlled manner to create a non-isotropic liquid mixture and then crosslinking of this mixture follows. An example of this is the buoyancy approach by Li et al., which should be suitable for generating hydrogels with well-defined compositional, mechanical, or biochemical gradients [24]. This simple method can be applied when (liquid) hydrogel precursors of different density exist or can be prepared using a suitable modifier. Firstly, the denser liquid is added to some mold as a base layer. Secondly, the lighter precursor is injected into the base layer (the authors used injection by an electronic dispenser). Buoyancy drives the formation of a continuous gradient throughout the whole liquid volume. Hydrogels prepared by buyoncy-driven gradient casting are shown in Figure 7 and Figure 8.

The initial gradient is preserved by subsequent gelation. The gradient can be controlled by the injection rate and densities of the liquid precursors. For example, the authors used agarose hydrogel of a fixed concentration with different concentrations of sucrose as density modifier.

The technique is not limited to hydrogels and was demonstrated by the authors on a range of different materials including polymers, liposomes, extracellular vesicles, etc. This versatile method is realizable via the use of standard laboratory apparatus.

## 3. Conclusions

The synthesis of gradient hydrogels represents an effort to advance hydrogel materials from simple pristine networks to hydrogels with designed and controlled supramolecular architectures with specific and smart properties. A variety of preparation methods are available—all technically realizable in the laboratory. Due to the variety of potential gradients and specific merits of each group of techniques it is impossible to select “a winner” even for a specific application. Some techniques like controlled mixing are realizable in any common laboratory, other require special facilities (for example 3D printing or electro-based techniques). Upscaling to industrial production still remains a challenge, however. Therefore, gradient hydrogels will probably remain special materials produced in relatively small batches—that is, they will be the domain of small-scale production units. The main areas of application are in biomimetics, tissue engineering, biomedicine, and environmental technologies. There is still a lack of information on some properties of gradient hydrogels—for example, their transport properties; there has been little experimental testing and investigation of diffusion gradients and their significance especially in bio-related applications. Another unexplored area is the computer modeling and design of gradient hydrogels, either on the basis of continuum mechanics and thermodynamics or the use of molecular modeling approaches.
